# Proteomic dataset of MECP2-deficient and wild-type human brain organoids under spaceflight and ground conditions

**DOI:** 10.1038/s41597-026-06881-5

**Published:** 2026-02-19

**Authors:** Aline M. A. Martins, Diogo G Biagi, Blake L. Tsu, Juliana de Saldanha da Gama Fischer, Luisa Bulcao Vieira Coelho, Paulo Costa Carvalho, Alysson R. Muotri

**Affiliations:** 1 Integrated Space Stem Cell Orbital Research Center, University of California San Diego, La Jolla, CA USA; 2 Laboratory for Structural and Computational Proteomics, Carlos Chagas Institute – Fiocruz Paraná, Curitiba, Brazil

## Abstract

This dataset contains mass spectrometry-based proteomic profiles of human brain organoids cultured on Earth for 30 days, then maintained aboard the International Space Station (ISS) for an additional 30 days, with matched ground controls that remained on Earth for the equivalent duration. Brain organoids were derived from induced pluripotent stem cell (iPSC) lines: Q83X, carrying a nonsense mutation in MECP2 from a male patient with Rett syndrome, and WT83, derived from the patient’s unaffected familial control. Rett syndrome is a severe X-linked neurodevelopmental disorder caused by loss-of-function mutations in MECP2, which encodes Methyl-CpG-binding protein 2, a critical epigenetic regulator. The spaceflight experiment was conducted using cryovials with automated control maintenance. Deep proteome coverage with approximately 6,000 protein groups was inferred from 56,639 peptides. This dataset provides unique insights into how the space environment affects human neural tissue and MECP2-related pathologies, serving as a resource for understanding spaceflight-induced neurological changes and as a steppingstone for future space missions.

## Background & Summary

Rett syndrome is a severe X-linked neurodevelopmental disorder caused by loss-of-function mutations in *MECP2*, which encodes Methyl-CpG-binding protein 2 (MeCP2)^1^, a critical epigenetic regulator that binds methylated DNA and functions as a repressor of L1 retroelements^2^. Affected individuals, albeit apparently developing normally, begin to show symptoms at very early ages, somewhere between 6–18 months, followed by progressive loss of acquired skills. This developmental trajectory suggests that pathological changes begin during early brain development, prior to the appearance of clinical symptoms. Despite affecting approximately 1 in 10,000 live female births, no curative treatments exist, and the mechanisms linking *MECP2* loss to early neurological dysfunction remain poorly understood.

Brain organoids derived from patient-specific induced pluripotent stem cells (iPSCs) have emerged as powerful three-dimensional reductionist models that recapitulate key features of human brain development and enable mechanistic investigation of neurodevelopmental disorders in controlled environments^3^. For Rett syndrome in particular, organoids offer distinct advantages: they capture distinct features of this disorder, allowing observation of how MECP2 loss disrupts maturation in real-time^4–6^. Unlike 2D neuronal cultures, organoids preserve the three-dimensional cytoarchitecture and diverse cell-type interactions critical for understanding MeCP2’s multifaceted role in neuronal maturation, synaptic development, and transcriptional regulation^7,8^.

Recently, it was shown that the space environment uniquely accelerates molecular and cellular processes that typically unfold gradually over months to years on Earth, creating what can be termed a “phenotypic compression” phenomenon^9^. This “temporal acceleration” is particularly valuable for investigating neurodevelopmental disorders, or conditions characterized by disrupted brain development during critical early-life windows. Disorders such as Rett syndrome, Autism Spectrum Disorder (ASD), Pitt-Hopkins syndrome, and other similar conditions manifest through aberrant developmental trajectories that are challenging to model in traditional laboratory settings due to the protracted timescales of human brain maturation. In this vein, our team has consistently conducted a series of experiments sending human brain organoids to the ISS aboard eight Commercial Resupply Services missions, including CRS-SpX18 and CRS-SpX30, each lasting approximately 30 days^10^. The space environment’s capacity to compress these developmental windows while maintaining cellular viability offers a unique experimental advantage to observe pathological mechanisms in weeks what would ordinarily require months or years of longitudinal culture. We utilized static experiments in 1 ml cryovials aboard the International Space Station, with automated thermal control, passive gas maintenance, and continuous environmental monitoring.

This data descriptor presents mass spectrometry-based proteomic profiles from brain organoids derived from the WT83 and Q83X human iPSC lines. All organoids underwent an initial 30-day differentiation period on Earth. Subsequently, they were divided into two groups: one was launched at the International Space Station (ISS), and the other was maintained on Earth as synchronous ground control. For the 30-day duration of the experiment, both groups were housed in identical, passive system modules. The Q83X line was derived from a male Rett syndrome patient carrying a nonsense mutation in exon 3 of *MECP2*^5^. This mutation changes a single DNA nucleotide from CAG (encoding glutamine) to TAG (stop codon) at position 83. Consequently, instead of producing the full-length 486-amino acid MeCP2 protein, translation terminates prematurely, yielding a severely truncated fragment (~17% of normal length) of only 82 aminoacids long that lacks critical functional domains for DNA binding, protein-protein interactions, transcriptional regulation, and chromatin remodeling. The WT83 control line was derived from first degree relative, an unaffected family member, providing control with same genetic background except for the disease-causing mutation.

The resulting dataset, comprising deep proteomic profiles with ~6,000 protein groups quantified across spaceflight and ground conditions in both genotypes, provides a unique resource for understanding how space environment and MECP2 loss interact to affect human neural development, with potential applications for astronaut health countermeasures and neurodevelopmental disorder therapeutics.

## Methods

### Organoid culture protocols

Human induced Pluripotent Stem Cells (iPSCs) were generated from skin biopsy-derived fibroblasts by reprogramming with the four canonical transcription factors (Klf4, Oct3/4, c-Myc, and Sox2)^6^. Two iPSC clones per individual were selected and karyotyped. The Q83X and WT83 iPSC lines used in this study were generated in the Muotri laboratory and have been previously characterized^8^; these lines are not commercially available but may be considered upon request to the corresponding authors. iPSCs were maintained on matrigel-coated dishes using mTeSR plus (STEMCELL Technologies). Brain organoids were generated using a semi-guided protocol developed in the Muotri lab^11^. Briefly, for neural induction, 3−4 million dissociated iPSCs per well were maintained in a 6-well low-attachment plate at 37 °C, 5% CO2, and at 95 rpm agitation. Then, cells were cultured for seven days in STEMdiffTM Neural Induction Medium (NIM) (STEMCELL technologies). For the neural maturation, we used supplemented media with 10 ng/mL of BDNF, 10 ng/mL of GDNF, 10 ng/mL of NT-3 (PeproTech), 200 uM of L-ascorbic acid and 1 mM dibutyryl-cAMP (Sigma-Aldrich) for seven days, with media changes every three days. At least two biological replicates were used in each one of the biological scenarios (WT83 and Q83X). Skin biopsies and fibroblast samples were obtained under UCSD IRB/ESCRO (University of California San Diego Institutional Review Board/Embryonic Stem Cell Research Oversight) protocol #141223 (‘Modeling genetic disorders using stem cells’), which approved the study and the sharing of data. Written informed consent for participation and open publication of de-identified data was obtained from all donors or, where applicable, from parents or legal guardians of pediatric participants. All procedures involving human material were conducted in accordance with the ethical principles of the Declaration of Helsinki and the Belmont Report.

### Spaceflight experimental setup

The Space Tango High-Throughput Passive System is a research platform designed for conducting numerous small-scale, static biological and physical experiments in the unique environment of microgravity aboard the International Space Station (ISS)^9^. This system is engineered to accommodate up to 588 individual samples housed within standard 1 mL cryovial tubes. The cryovials are housed within Space Tango’s Powered Ascent Utility Locker (PAUL), which maintains a controlled atmosphere at 37 °C and 5% CO2 throughout the mission. Each cryovial cap contains a gas-permeable polydimethylsiloxane (PDMS) membrane, enabling passive diffusion of O2 and CO2 between the PAUL atmosphere and the culture medium inside each vial. Constant environmental monitoring tracks all relevant parameters, ensuring experimental integrity and traceability. Samples are first loaded into pre-labeled cryovials, which are then securely capped to prevent any contamination or leakage. Finally, these cryovials are strategically placed into specifically mapped locations within designated containment blocks, guaranteeing that every sample can be tracked precisely from Earth to orbit and back.

Prior ground validation experiments confirmed that organoids from the cell lines used in this study can survive 30 days in static culture without media exchange. Specifically, WT83 organoids maintained in 1 mL cryovials at 37 °C for one month showed sustained metabolic activity as measured by glucose consumption and acceptable cellular stress levels assessed by lactate dehydrogenase (LDH) release^12^.

### Proteomics sample preparation

Proteins were extracted using 8 M urea prepared in 100 mM triethylammonium bicarbonate (TEAB) buffer. Cell lysis was achieved by two cycles of sonication on ice, each lasting 20 seconds at 60% amplitude. Protein concentration was determined using the bicinchoninic acid (BCA) assay. For each sample, 50 µgs of total protein was reduced by 5 mM Tris(2-carboxyethyl) phosphine hydrochloride (TCEP) for 30 minutes at room temperature, followed by alkylation with 15 mM chloroacetamide for 30 minutes in the dark at room temperature. Proteins were then digested overnight (18 hours) at 37 °C with sequencing-grade modified trypsin (Promega, V511A) at an enzyme-to-substrate (E:S) ratio of 1:50 (w/w). Digestion was quenched by acidification with formic acid (FA) to a final concentration of 1%. Samples were centrifuged at 18,000 × g for 10 minutes to remove insoluble debris. Peptides were subsequently desalted using C18 Stage Tips according to the protocol described by Rappsilber *et al*.^13^.

Data was acquired using a Thermo Scientific Vanquish Neo UHPLC system coupled to a Thermo Scientific Orbitrap Astral mass spectrometer equipped with an EASY-spray source. Sample concentrations were normalized to 100 ng/uL with an injection volume of 4uL. The Vanquish Neo was configured to a “trap-and-elute” workflow. Sample loading utilized combined pressure and flow rate controls where the maximum pressure was set to 800 bar and the flow rate capped at 200 μL/min. Samples were loaded and de-salted on a Thermo Scientific PepMap Neo C18 300 um × 5 mm(PN: 174500) trap cartridge prior to separation. Washed peptides are then eluted off the trap and separated on a Thermo Scientific EASY-Spray 75 um × 15 cm, 2um(PN: ES75150PN) heated to 50 °C. Separation was performed over a 60 min gradient, with the active gradient starting from 4% to 35% B with flow rates starting at 900nL/min for the first 20 mins then decreased to 300nL/min for 37 mins, this is then followed by 3 mins of column washing at 500nL/min at 95%B. Mobile phase A was 0.1% formic acid in water and mobile phase B was 80% acetonitrile and 0.1% formic acid in water.

Orbitrap Astral acquisition mode was set to data-dependent acquisition (DDA) in positive polarity. Full scans were acquired in the Orbitrap at 240,000 resolution with a scan range of 350–1400 *m/z*. AGC targets were set to 200% with a maximum injection time of 5 ms. Monoisotopic peak selection (MIPS), intensity, charge state, and dynamic exclusion filters were used to trigger data-dependent MS2 scans. Monoisotopic peak selection setting was set to peptide. The minimum precursor intensity threshold was set to 5.0e3. Peptides with charge states 2 + through 5 + were selected. Dynamic exclusion excluded precursors 1 time for a 30 s duration with low and high mass tolerances both set at 10ppm. ddMS2 scans were acquired in the Astral analyzer with a mass range of 150–2000 *m/z*. Isolation window was set to 2 *m/z* with no isolation offset. Normalized HCD collision energy was set to 29%. AGC targets were set to standard with a maximum injection time of 3.5 ms.

### Data processing pipelines

Peptide identification and protein inference were carried out using PatternLab for Proteomics V as described in its bioinformatics protocol^14^. Briefly, this software is freely available for academic use at http://www.patternlabforproteomics.org. Sequences for *Homo sapiens* were downloaded from UniProt (Swiss-Prot + TrEMBL) on April 16^th^, 2025. A target-decoy database was created to include both reversed sequences and 104 common mass spectrometry contaminants, excluding keratin, which was not considered a contaminant in this analysis. The Y.A.D.A. 3.0 deconvolution algorithm was used for preprocessing the data, allowing for multiplexed spectra identification^15^. The Comet search engine^16^ was employed for mass spectra identification, considering fully and semi-tryptic peptide candidates with masses ranging from 500 to 6000 Da, allowing for up to two missed cleavages, with a precursor mass tolerance of 35 ppm and MS/MS bins of 0.02 *m/z*. Only peptide candidates with six or more aminoacids were considered. The search parameters included carbamidomethylation of cysteine as a fixed modification and oxidation of methionine as a variable modification.

### Validation PSM

The validity of the PSMs was performed using the Search Engine Processor (SEPro)^17^. The identifications were categorized by charge state (2+ and ≥3+) and tryptic status, creating four distinct subgroups. For each subgroup, XCorr, DeltaCN, DeltaPPM, and Peaks Matches values were utilized to develop a Bayesian discriminator. Identifications were then sorted in nondecreasing order based on the discriminator score. A cutoff score was established to accept a false discovery rate (FDR) of 1% at the protein level, determined by the number of decoys^18^. This process was independently applied to each data subset, ensuring an FDR that was independent of charge state or tryptic status. After that, proteins with score below 2 or identifications deviating by more than 10 ppm from the theoretical mass were excluded. This final filter resulted in protein-level FDRs below 1% for all search results.

## Data Record

The mass spectrometry proteomics data are available at the PRIDE repository via ProteomeXchange with the dataset identifier PXD069807^19^. The repository contains the following files:

### Raw files

Ten Orbitrap Astral mass spectrometry raw files corresponding to the biological replicates described in Table 1.

**Table 1:** Summary of proteomic identifications for brain organoid samples cultured on ground and ISS conditions.

Label	*Condition*	Organoid	Spectra	Peptides	Proteins	PGroups
Tube 26	*Ground*	QX	103621	24490	15331	3495
Tube27	Ground	QX	88952	20926	14436	3267
Tube28	Ground	QX	112570	27061	15897	3614
Tube32	Ground	WT83	119937	28858	16725	3808
Tube33	Ground	WT83	108098	25443	16127	3671
Tube34	Ground	WT83	114170	26859	16562	3759
Tube74	ISS	QX	102027	24416	15075	3497
Tube75	ISS	QX	104524	24910	15241	3504
Tube80	ISS	WT83	117975	30481	17732	4051
Tube81	ISS	WT83	97481	23091	15337	3526

Each row represents an individual biological replicate analyzed by LC-MS/MS. Label indicates the number in the sample tube; this number is also available in the mass spectrometry raw file. Condition specifies whether the organoid was maintained on the ground (Ground) or aboard the International Space Station (ISS) during the 30-day experimental period following the initial 30-day ground culture. Organoid denotes the iPSC line used: QX (Q83X, MECP2-deficient Rett syndrome model carrying the Q83X nonsense mutation) or WT83 (isogenic control). Spectra, Peptides, and Proteins refer to the number of confident identifications after applying the statistical filters. The Proteins column reflects the total number of protein database entries matched by peptides. The PGroups (Protein Groups) column, in contrast, reports the minimal set of proteins that explain all identified peptides according to the principle of maximum parsimony.

### Search results

PatternLab for Proteomics project files (.sepr2) containing peptide-spectrum matches and protein identifications at 1% FDR, organized by condition (Ground_QX.sepr2, Ground_WT83.sepr2, ISS_QX.sepr2, ISS_WT83.sepr2).

The protein identification and quantitation spreadsheet is available at Zenodo^20^. This file contains all protein groups with corresponding spectral counts and quantitation values for each biological replicate, with columns for protein accession, description, and per-sample quantitation.

## Technical Validation

### Protein identification

Each mass spectrometry raw file is identified by its corresponding tube number (e.g., Tube27.raw, Tube28.raw), which matches the sample labels provided in the metadata. The dataset comprises nine LC-MS/MS acquisitions representing biological replicates from two genotypes (QX and WT83) cultured under two environmental conditions (Ground and ISS).

Across all samples, a total of 56,639 peptides were confidently identified using PatternLab for Proteomics 5.1 at 1% FDR. These peptide sequences map to 21,161 individual protein entries in the UniProt sequence database (*Homo sapiens*, Swiss-Prot + TrEMBL, downloaded April 16th, 2025). Applying the principle of maximum parsimony, selecting the minimum number of protein entries necessary to explain all identified peptides, yields 5,952 protein groups across the entire dataset. A summary of the sample metadata and per-replicate identification statistics is presented in Table 1. The complete protein identification and quantitation data for each of the files in Table 1 are available in the accompanying spreadsheet deposited in Zenodo^20^.

### Validation of the rett syndrome genotype at the protein level

**Fig. 1:**
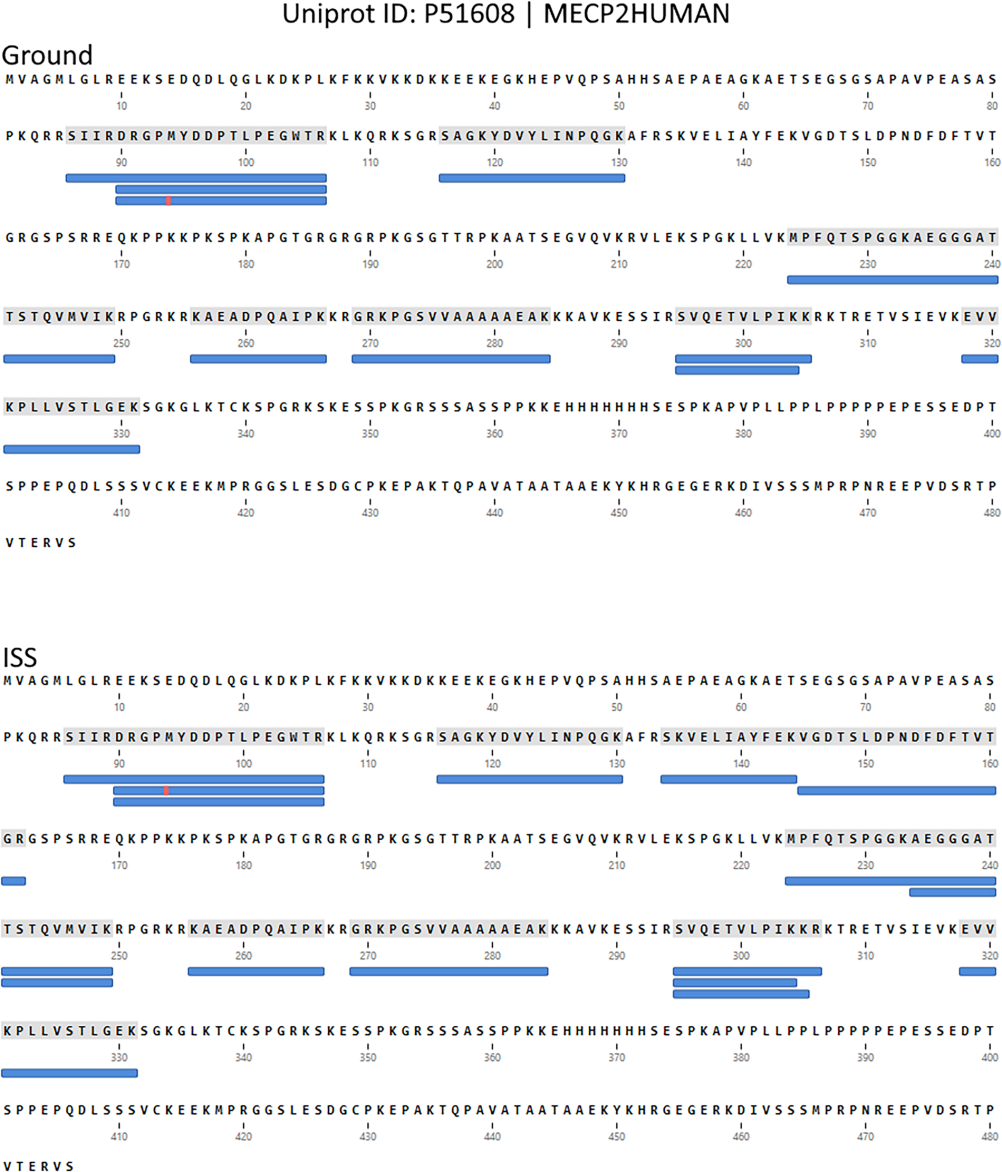
Proteomic validation of the MeCP2 protein in WT83 brain organoids. The figure displays a peptide coverage map for the full-length MeCP2 protein (UniProt: P51608) identified in wild-type (WT83) samples. Blue bars indicate the sequences of confidently identified peptides mapped to the protein. The red stripe on a peptide indicates a post-translational modification (oxidized methionine). Notably, no peptides corresponding to MeCP2 were detected in any of the MECP2-deficient (QX) samples, confirming a true loss-of-function at the protein level.

To validate our Rett syndrome model at the protein level, we examined the peptide evidence for MeCP2 (UniProt: P51608). In all WT83 replicates from both ground and ISS conditions, we observed peptides spanning the full length of the protein, this providing multiple independent evidences of the expression and translation of wild-type MeCP2 (Fig. 1). In stark contrast, no peptides corresponding to MeCP2 were detected in any of the QX samples. This complete absence of the protein, decurrent from the Q83X nonsense mutation, could likely be due to degradation of the mutant mRNA transcript via nonsense-mediated decay (NMD) before translation can occur^21^. This result confirms the loss-of-function and validates the Rett syndrome genotype in our organoid model.

### Protein identification and chromatographic reproducibility

**Fig. 2:**
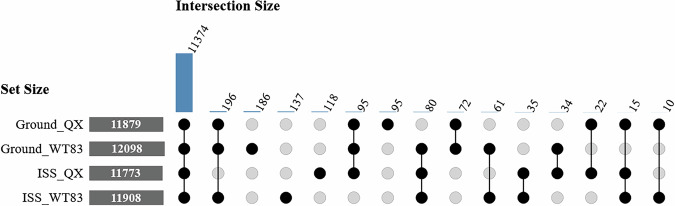
Overlap of identified protein groups across experimental conditions. The Upset plot shows the number of shared protein groups between the four conditions, indicated by the vertical bars. The vast majority of proteins entries (11,374) form a core proteome found in all samples. The bars on the left show the total protein groups identified per condition.

To assess the quality and reproducibility of the dataset, we first evaluated the consistency of protein identifications across all conditions. An Upset plot of identification found in 2 or more analyses (Fig. 2) revealed a substantial core proteome of 11,374 entries common to all four conditions, demonstrating high consistency and reproducibility in proteome coverage. Beyond this shared core, the plot also highlights biologically relevant subsets, such as, for example, proteins only identified in the space condition and others only in ground.

**Fig. 3:**
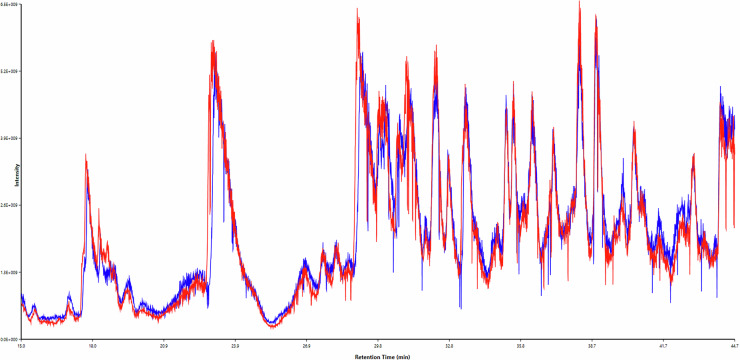
Chromatographic profile overlay. Total ion chromatograms from two representative samples (QX tube 26, red; tube 27, blue) showing high similarity in peptide elution patterns across the LC-MS/MS runs.

**Fig. 4:**
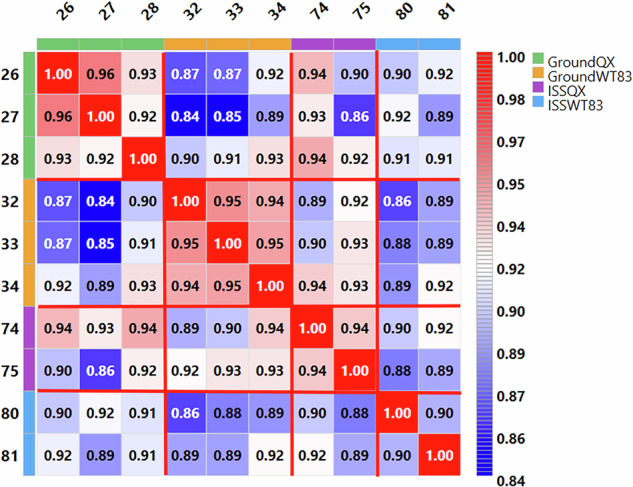
Heatmap showing Pearson correlation coefficients between total ion chromatograms (TIC) from all ten brain organoid samples analyzed by LC-MS/MS on the Orbitrap Astral. Sample numbers (tube identifiers as per Table 1) are displayed on rows and columns. Color-coded annotation bars indicate experimental conditions: Ground-QX (MECP2-deficient ground control, green), Ground-WT83 (wild-type ground control, orange), ISS-QX (MECP2-deficient spaceflight, purple), and ISS-WT83 (wild-type spaceflight, light blue). TICs were aligned using DTW to account for retention time shifts, and Pearson correlations were computed on the aligned traces. The color scale represents correlation values from 0.84 (blue, lower correlation) to 1.00 (red, perfect correlation). Heatmap was generated using the ChromaCompare module in PatternLab for Proteomics 5.1.

To complement this analysis, we then examined the chromatographic similarity across samples. Figure 3 shows an overlay comparison of two representative chromatographic runs (QX tube 26 and tube 27), demonstrating the high degree of similarity in chromatographic profiles between samples. A comprehensive heatmap of Pearson correlations between the chromatograms of all ten samples provides a global overview of the chromatographic similarity across the entire experiment (Fig. 4). The Upset plot, chromatographic overlays, and correlation heatmap were generated using the ‘Overlap Analysis’ and ‘ChromaCompare’ modules, both available within PatternLab 5.1.

## Usage Notes

This dataset represents a unique resource for the scientific community, generated under exceptional constraints inherent to spaceflight research with elevated costs. The biological material was collected during a 30-day mission aboard the ISS, which imposed strict limitations on sample capacity due to the need to accommodate additional concurrent experiments within the confined orbital laboratory environment. These logistical and spatial constraints severely restrict the number of biological replicates that can be obtained in a single mission, making each sample valuable. Importantly, this dataset serves as a foundational reference for ongoing follow-up missions that will expand the biological replicate pool over time, collectively building a comprehensive understanding of how the spaceflight environment affects human neural tissue development and neurodevelopmental disease phenotypes.

Despite the limited number of biological replicates from this initial mission, the dataset enables several high-impact applications:

### Establishing baseline molecular signatures for spaceflight neuroscience and informing countermeasures for long-duration space missions

As humanity advances toward long-duration missions such as to Mars and beyond, understanding how the space environment affects neural tissue at the molecular level becomes critical for astronaut health. The ~6,000 protein groups quantified here establish baseline signatures of spaceflight-induced changes in human neural tissue, as such, this dataset can be used to reveal key proteomic alterations for these conditions allowing development of targeted countermeasures. This information is essential for ongoing and future missions that use neural 3D culture experiments for disease modelling.

### Comparative molecular dissection of MECP2-deficient *versus* wild-type neural development under spaceflight conditions

The pairing of Q83X (MECP2-deficient) and WT83 (wild-type control) organoids provides a powerful framework for dissecting how loss of MeCP2 function alters the neural proteome response to spaceflight stress. By comparing proteomic profiles between genotypes under both ground and spaceflight conditions, this dataset will aid studies that investigate MECP2-dependent *versus* MECP2-independent molecular responses to space environment. This four-way comparison (genotype × environment) will enable the discovery of pathways that are specifically dysregulated in Rett syndrome contexts, as a disease modelling accelerated by the space environment, potentially revealing therapeutic targets that could normalize disease-associated molecular signatures.

### Investigation of accelerated neurodevelopmental phenotypes and L1 retroelement biology

The “phenotypic compression” observed in spaceflight conditions, where molecular and cellular processes that typically unfold over months on Earth are accelerated, creates a unique temporal window for studying neurodevelopmental trajectories. This dataset captures proteomic states that may represent accelerated aging or stress-induced alterations in neural development, providing a reference for identifying proteins involved in developmental timing and stress response. Additionally, given MeCP2’s established role as a repressor of L1 retroelements, this dataset offers opportunities to investigate whether spaceflight conditions—known to induce DNA damage and cellular stress—interact with MECP2 deficiency to affect L1 retroelement control^2^. Such investigations could reveal novel links between environmental stress, epigenetic regulation, and transposable element activity in human neural tissue, with implications for understanding both spaceflight-induced genomic instability and neurodevelopmental disorders characterized by epigenetic dysregulation.

## Data Availability

The mass spectrometry proteomics data have been deposited to the ProteomeXchange Consortium via the PRIDE partner repository with the dataset identifier PXD069807.

## Code availability

In this study, no custom code was utilized. The software used for protein identification and generating all displayed images and analyses, PatternLab for Proteomics V^14^, is freely available for academic use at http://patternlabforproteomics.org/51.
